# Left gastric vein-based noninvasive test for esophageal varices: a same-day comparison of portal hemodynamic assessment with endoscopic appearance

**DOI:** 10.1038/s41424-018-0021-8

**Published:** 2018-05-25

**Authors:** Hitoshi Maruyama, Kazufumi Kobayashi, Soichiro Kiyono, Sadahisa Ogasawara, Yoshihiko Ooka, Eiichiro Suzuki, Tetsuhiro Chiba, Naoya Kato

**Affiliations:** Department of Gastroenterology, Chiba University Graduate School of Medicine, 1-8-1, Inohana, Chuo-ku, 260-8670 Japan

## Abstract

**Objective:**

To examine the effect of hemodynamic assessment of the left gastric vein (LGV) as a noninvasive test to diagnose esophageal varices (EV) in cirrhosis patients.

**Methods:**

This cross-sectional study consisted of 229 cirrhosis patients (62.7 ± 11.8 years; Child-Pugh score 5–14). One hundred fifty-four patients had EV (67.2%; small, 53; medium, 71; large, 30). All patients underwent a blood test and Doppler ultrasound followed by upper gastrointestinal endoscopy on the same day. The diagnostic ability for EV was compared between LGV-related findings and the platelet count/spleen diameter ratio (Plt/Spl).

**Results:**

The detectability of the LGV was higher in patients with EV (129/144, 89.6%) than in those without (35/75, 46.7%; *p* < 0.0001), and was higher in those with large EV (30/30, 100%) than in those without (134/199, 67.3%; *p* = 0.0002). The positive detection of the LGV showed 100% sensitivity and negative predictive value (NPV) to identify large EV in the whole cohort and compensated group (*n* = 127). The best cutoff value in the LGV diameter was 5.35 mm to identify large EV, showing 0.753 area under the receiver operating characteristic curve (AUROC) with 90% sensitivity and 96.5% NPV. The Plt/Spl showed 62.1% sensitivity and 87.1% NPV, and the best cutoff value was 442.9 to identify large EV with 0.658 AUROC, which was comparable to LGV-based assessment (*p* = 0.162).

**Conclusions:**

This same-day comparison study demonstrated the value of LGV-based noninvasive test to identify large EV with high sensitivity and NPV in cirrhosis patients at a lower cost.

## Introduction

Portal hypertension is the principal pathogenesis of cirrhosis. The underlying mechanisms consists of increased portal inflow and/or outflow resistance, and are indirectly affected by the development of intra-/extra-hepatic collateral vessels^1^. Taken together, it may result in various complications in patients with cirrhosis, gastroesophageal varices, portal hypertensive gastropathy (PHG), ascites, and hepatic encephalopathy^2^.

Esophageal varices (EV) represents a major hemodynamic abnormality of cirrhosis. Investigators have shown a 30–40% frequency in compensated cirrhosis patients, and 60% in patients with ascites^3^. A newly developed varices may be detected in ~5–10% per year of cirrhosis patients with no sign of varices^4^. Furthermore, the overall bleeding incidence from EV is ~25% in 2 years^5^, and the mortality rate from EV bleeding is ~20%, even with the recent improvements in the clinical management^6^.

Noninvasive markers to diagnose EV have gained attention since the report of the platelet count/spleen diameter ratio (Plt/Spl) by Giannini et al^7^. However, the diagnostic abilities of the noninvasive tests to detect and to grade EV are still insufficient, although a number of studies have been performed^8–12^.

The left gastric vein (LGV) is a major pathway that brings blood flow into the EV via the upper stomach. A previous study reported that the velocity in the LGV is associated with the development of EV and bleeding risk^13^. However, as it was performed in the early 1990s, a recent ultrasound (US) equipment with much improved sensitivity and signal-to-noise ratio may provide a more detailed evaluation of the LGV hemodynamics, which may have the potential to diagnose EV noninvasively. The aim of this cross-sectional study was to examine the effect of the physiological hemodynamic assessment of the LGV, which is a specific vascular route to EV anatomically and hemodynamically, as a noninvasive test to identify EV in patients with cirrhosis in a same-day comparison setting.

## Methods

### Study

This is a newly designed cross-sectional study performed between September 2011 and September 2017 in our university hospital after obtaining informed written consent from all participants. It was approved by the ethical committee of our department as having an appropriate design for publication (C-U 419). The potential participants of the study were the following patients: (1) those who were diagnosed with cirrhosis by a laboratory test combined with two different imaging modalities, US and computed tomography (CT)/magnetic resonance imaging (MRI) in the outpatient clinic of our department, (2) those with no history of EV treatment. In addition, the study excluded the following patients: (1) those with portal vein thrombus (partial/complete), cavernoma, portal vein tumor thrombus, or intrahepatic arterioportal shunt, all diagnosed by the radiological imaging, (2) those with a history of abdominal surgery, partial splenic embolization, or transjugular intrahepatic portosystemic shunt, (3) those with advanced hepatocellular carcinoma stage C/D by the Barcelona-Clinic Liver Cancer Staging System^14^, (4) those patients who were pregnant at the time of the study.

The collecting of blood samples, Doppler US, and upper gastrointestinal endoscopy were scheduled on the same day for the study participants. Because the Doppler US was performed before the endoscopic examination, the US operator was blinded to the results of endoscopic findings.

The degree of ascites was defined by the recent guideline^15^. A compensated cirrhosis was defined by the following criteria to classify the patients independently associated with the presence of EV: those without ascites, icterus (total bilirubin > 3.0 mg/dL), or overt hepatic encephalopathy assessed according to the literature^16^. The Plt/Spl was also calculated using the data on the same day^7^, and the analysis was performed by using the cutoff value of 909 presented by the literature^7^ or that proposed by the present study.

Endoscopic examination was performed by KK or SK, and gastroesophageal varices and PHG were evaluated according to the general rules of the Japan Research Society for Portal Hypertension^17^, being assessed independently with respect to the US findings.

### Ultrasound

US examination was performed with the patients in the supine position after fasting for 10 h or more, using an SSA-770A or 790 A (Toshiba, Tokyo, Japan) with a 3.75 MHz convex probe. The portal system was carefully observed, and the collateral vessels were identified according to the literature^18^. The maximum diameter of the portal trunk and the LGV was measured, and the blood flow was assessed using the pulsed Doppler method with the sampling point at the width corresponding to the diameter of the vessel and at an angle below 60 degrees between the US beam and the vessel^18^.

The LGV detection was defined by the successful demonstration of the vessel on the B-mode image^13^ with both positive intravascular color signal under the optimal setting of color gain and velocity range and positive intravascular blood flow on the pulsed Doppler image. The mean velocity (cm/s) was measured, and the mean flow volume (mL/min) was calculated by multiplying the mean velocity for 1 s for the cross-section of the vessel, and multiplying it by 60 s.

Spleen size (mm^2^) was also calculated by multiplying the distance from the splenic hilum to the caudal polar angle, measured with two intersecting lines. The upper limit of normal used in the study was 2000 mm^2^^18^. The data used for analysis were the average values, calculated using measurements taken 2–4 times. All of the US examinations were performed by HM, who had more than 20 years of US experience.

### Statistical analysis

The study used Student’s *t*-test, analysis of variance, or the Pearson product-moment correlation coefficient for continuous variables, and Fisher’s exact test or chi-square test for categorical variables. The diagnostic abilities for detecting/grading EV were assessed by the receiver operating characteristic (ROC) curve, and the area under the ROC curve (AUROC), 95% confidence interval (CI), sensitivity, specificity, positive predictive value (PPV), negative predictive value (NPV), accuracy and best cutoff value were calculated. The probability values < 0.05 were considered to be statistically significant. The statistical values were calculated using SAS software (SAS Institute Inc., Cary, NC, USA).

## Results

### Patient characteristics

There were 229 cirrhosis patients in the study (male 134, female 95; age 62.7 ± 11.8, 18–87; Table 1). One hundred fifty-four patients had EV (67.2%), as follows: small, 53 (23.1 %); medium, 71 (31 %); and large, 30 (13.1 %). The Child-Pugh score ranged from 5 to 14 (7.2 ± 2.0).

**Table 1:** Patient characteristics

Number	229
Age	62.7 ± 11.8(18–87)
Sex (male/female)	134 (58.5%)/95 (41.5%)
Body mass index (kg/m^2^)	21.1 ± 9.4 (14.5–53.5)
Etiology(HCV/HBV/HBV+HCV/alcohol/NASH/PBC/AIH/PBC+AIH/PSC/NBNC)	71 (31%)/14 (6.1%)/1 (0.4%)/50 (21.9%)/31 (13.5%)/20 (8.7%)/7 (3.1%)/2 (0.9%)/3 (1.3%)/30 (13.1%)
Esophageal varices, *n* (%)	
None	75 (32.8%)
Small	53 (23.1%)
Medium	71 (31%)
Large	30 (13.1%)
Gastric fundal varices (None/small/medium/large)	158 (69%)/22 (9.6%)/31 (13.5%)/18 (7.9%)
Portal hypertensive gastropathy (−/+)	171 (74.7%)/58 (25.3%)
Ascites (−/mild/moderate to severe)	149 (65.1%)/57 (24.9%)/23 (10%)
Splenomegaly (−/+)	68 (29.7%)/161 (70.3%)
Hepatocellular carcinoma	175 (76.4%)/54 (23.6%)
Blood test	
Platelet count (×10^9^/L)	89.6 ± 49(77–300)
Aspartate aminotransferase (IU/L)	53.1 ± 44(3–420)
Alanine aminotransferase (IU/L)	34.9 ± 37.8(2–501)
Albumin (g/dL)	3.3 ± 0.7 (1.0–4.7)
Total bilirubin (mg/dL)	2.0 ± 2.6 (0.1–22.6)
Prothrombin time (%)	75.5 ± 20.6 (5–125)
Plt/Spl^a^	767.1 ± 530.2 (44.2–3180)
Child-Pugh score	7.2 ± 2.0 (5–14)
Child-Pugh classification (A/B/C)^b^	98 (42.8%)/98 (42.8%)/28 (12.2%)

Data are expressed as number or mean ± standard deviation (percentage or range)

*AIH* autoimmune hepatitis, *HBV* hepatitis B virus, *HCV* hepatitis C virus, *HBV* hepatitis B virus, *NASH* non-alcoholic steatohepatitis, *NBNC* non B non C, *PBC* primary biliary cholangitis, Plt/Splplatelet count/spleen diameter ratio, *PSC* primary sclerosing cholangitis

^a^ Calculated by the formula, platelet count/spleen bipolar diameter

^b^ Five patients were excluded because of taking warfarin

### Detectability of the LGV

The LGV was successfully detected in 164/229 patients (71.6 %), with forward direction in 12, bidirectional in 15, and reverse direction in 137 (Table 2). The body mass index (BMI) (kg/m^2^) was higher in the undetected group (27.0 ± 6.7) than in the detected group (23.3 ± 3.7, p = 0.0003). However, detectability of the LGV was not affected by the degree of ascites, with 104/149 (69.8%) in patients without ascites, 44/57 (77.2%) in those with mild ascites, and 16/23 (69.6%) in those with moderate/severe ascites (*p* = 0.625). The detectability of the LGV also was not affected by the severity of liver function, with 71/98 (72.4%) in Child A, 73/98 (74.5%) in Child B, and 18/28 (64.3%) in Child C (*p* = 0.6).

**Table 2:** Measurement data in the portal vein and the left gastric vein

	Flow direction (Forward/bidirectional/reverse)	Diameter (mm)	Velocity (cm/s)	Flow volume (mL/min)
Portal vein (*n* = 229)	209 (91.3%)/15 (6.5%)/5 (2.2%)	10.9 ± 2.0 (2.7–16.3)	12.4 ± 2.9^b^ (6.3–22.5)	741.5 ± 277.8^b^ (255–1970)
Left gastric vein (*n* = 164)^a^	12 (7.3%)/15 (9.1%)/137 (83.6%)	5.4 ± 1.7 (0.9–11.6)	12.4 ± 3.5^c^ (5.2–25.8)	220.8 ± 174.5^c35–1124^

Data are expressed as number or mean ± SD (percentage or range).

^a^ Left gastric vein was not detected in 65 patients

^b^ Velocity and flow volume in the portal vein showing forward flow direction

^c^ Velocity and flow volume in the left gastric vein showing reverse flow direction

The detectability of the LGV was higher in patients with EV (129/144,　89.6%) than in those without (35/75, 46.7%; *p* < 0.0001), and was higher in those with large EV (30/30, 100%) than in those without (134/199, 67.3%; *p* = 0.0002). However, the BMI showed no difference between patients with EV (23.9 ± 4.3) and those without (25.2 ± 6.2. *p* = 0.1), and between patients with large EV (23.3 ± 3.6) and those without (24.5 ± 5.1, *p* = 0.23).

The EV was present in 25/65 patients (38.5 %; small, 17; medium, 8) with negative detection of the LGV. The detectability of the LGV was higher in patients with cardiac varices (82/96, 85.4%) than in those without (82/133, 61.7%; *p* < 0.0001), and in those with splenomegaly (122/161, 75.8%) than in those without (42/68, 61.8%; *p* = 0.032). However, a presence of gastric fundal varices (45/71 vs. 119/158, *p* = 0.065) or PHG (47/58 vs. 117/171, *p* = 0.066) was not a significant factor for LGV detection. In 65 patients with negative LGV detection, there were no significant differences in the clinical data between patients with EV (*n* = 25) and those without (*n* = 40).

### Hemodynamics in the LGV

In patients with positive LGV detection, reverse direction was significantly more frequent in patients with EV (113/129, 87.6%) than in those without (24/35, 68.6%; *p* = 0.007), and in those with large EV (30/30, 100%) than in those without (107/134, 79.9%; *p* = 0.007). However, the frequency of reverse direction was not affected by a presence of cardiac varices (72/82 vs. 65/82, *p* = 0.14) or splenomegaly (105/122 vs. 32/42, *p* = 0.14).

The diameter of the LGV ranged from 0.9 to 11.6 (5.4 ± 1.7) mm, and the velocity and flow volume in the LGV showing reverse direction were 5.2 to 25.8 (12.4 ± 3.5) cm/s and 35 to 1124 (220.8 ± 174.5) mL/min, respectively (Table 2). There were significant differences in the parameters between patients with and without large EV: diameter (6.4 ± 1.0 mm vs. 5.6 ± 1.7 mm, *p* = 0.004), and flow volume (265.4 ± 103.7 mL/min vs. 208.5 ± 188.0 mL/min, *p* = 0.035). The diameter, velocity, and flow volume in the LGV ranged from 2.6 to 10.9 mm, 5.2 to 19.25 cm/s, and 31 to 851 mL/min in patients with EV, and no EV was detected in patients who had an LGV with diameter greater than 10.9 mm, velocity greater than 19.25 cm/s, or flow volume greater than 851 mL/min.

### Diagnostic ability as a noninvasive test for EV

The positive detection of the LGV showed 83.8% sensitivity and 61.5% NPV to identify any EV, and 100% sensitivity and 100% NPV to identify large EV. The best cutoff value of the LGV diameter was 3.55 mm to identify any EV, showing 0.575 AUROC with 93% sensitivity and 52.6% NPV, and that was 5.35 mm to identify large EV, showing 0.753 AUROC with 90% sensitivity and 96.5% NPV (Table 3).

**Table 3:** The diagnostic ability to identify esophageal varices

	AUROC	95% CI	Cutoff value	Sensitivity (%)	Specificity (%)	PPV (%)	NPV (%)	Accuracy (%)	*P*-value vs. Plt/Spl
*Positive LGV detection*									
Any EV	—	—	—	83.8	53.3	78.7	61.5	73.8	—
Large EV	—	—	—	100	32.7	18.3	100	41.5	—
*LGV diameter* *(mm)*									
Any EV	0.575	0.451–0.700	3.55	93	28.6	82.6	52.6	79.1	0.232
Large EV	0.753	0.677–0.829	5.35	90	61.7	34.6	96.5	66.9	0.162
*LGV* *velocity* ^*a*^ *(cm/s)*									
Any EV	0.638	0.503–0.773	14.1	21.2	54.2	68.6	12.8	27.0	0.498
Large EV	0.601	0.485–0.716	12.55	60	58.9	29	84	59.1	0.39
*LGV* *flow* *volume* ^*a*^ *(mL/min)*									
Any EV	0.629	0.510–0.749	140	60.2	16.7	77.3	8.2	52.6	0.459
Large EV	0.706	0.605–0.808	160	90	52.3	34.6	94.9	60.6	0.377
*Plt/Spl*									
Any EV	0.646	0.537–0.755	535.7	54.6	82.6	93.8	27.5	59.4	—
Large EV	0.658	0.543–0.773	442.9	62.1	71.2	37.5	87.1	69.2	—

*AUROC* area under the receiver operating characteristic curve, *CI* confidence interval, *EV* esophageal varices, *LGV* left gastric vein, *NPV* negative predictive value, *Plt/Spl* platelet count/spleen diameter ratio, *PPV* positive predictive value

^a^ Velocity and flow volume in the left gastric vein showing reverse flow direction

To identify large EV in patients with LGV showing reverse direction, the best cutoff value of the velocity was 12.55 cm/s, showing 0.601 AUROC with 60% sensitivity and 84% NPV, and that of the flow volume was 160 mL/min, showing 0.706 AUROC with 90% sensitivity and 94.9% NPV (Table 3).

**Fig. 1:**
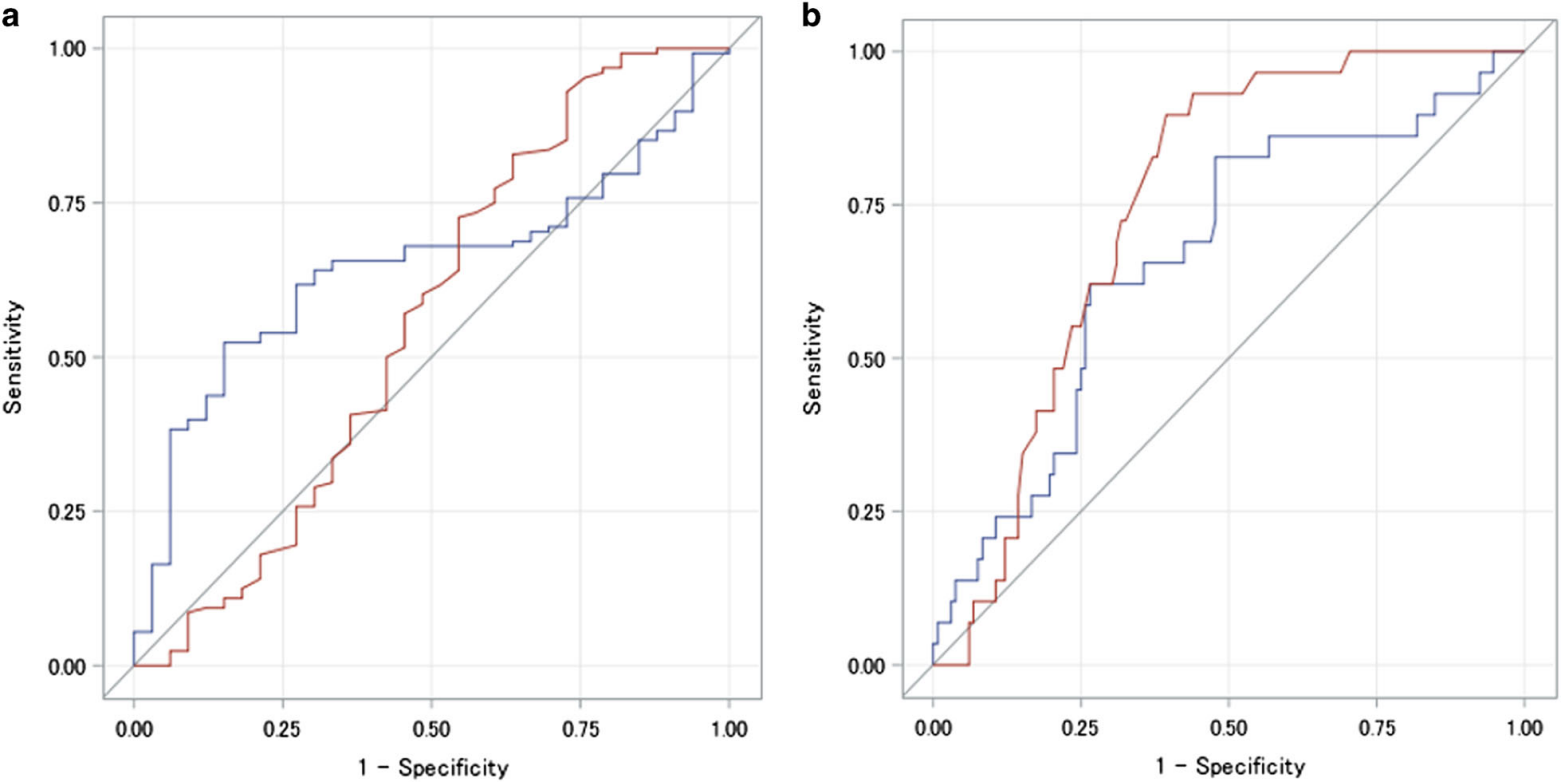
Comparison of receiver operating characteristic curve to diagnose esophageal varices. The AUROC showed no difference between LGV diameter-based assessment, which was the best in the LGV-related data, and the Plt/Spl-based assessment, for any esophageal varices (**a**, *p* = 0.232) and for large esophageal varices (**b**, *p* = 0.162). **a** To identify any esophageal varices. **b** To identify large esophageal varices. Blue line, Plt/Spl-based assessment; red line, LGV diameter-based assessment. AUROC the area under the receiver operating characteristic curve, Plt/Spl platelet count/spleen diameter ratio, LGV left gastric vein

The Plt/Spl with the cutoff value 909 provided 75.3% sensitivity, 37.3% specificity, 71.2% PPV, 42.4% NPV, and 62.9% accuracy to identify any EV, and 86.7% sensitivity, 31.2% specificity, 16.0% PPV, 93.9% NPV, and 38.4% accuracy to identify large EV. When the best cutoff value was determined in the present cohort, it was 535.7 to identify any EV, showing 0.646 AUROC with 54.6% sensitivity and 27.5% NPV, and was 442.9 to identify large EV, showing 0.658 AUROC with 62.1% sensitivity and 87.1% NPV (Table 3). The AUROC showed no difference between LGV diameter-based ability, which was the best in the LGV-related assessment, and the Plt/Spl-based assessment, for any EV (*p* = 0.232) and for large EV (*p* = 0.162; Fig. 1).

### Diagnostic abilities to identify large EV in compensated liver cirrhosis

There were 127 patients with compensated liver cirrhosis, and 11 patients had large EV. The positive detection of the LGV showed 100% sensitivity and 100% NPV, and the best cutoff value of the LGV diameter was 5.4 mm, showing 0.700 AUROC with 81.8% sensitivity and 96.2% NPV. In the cohort with LGV showing reverse direction, the velocity had 0.646 AUROC under the best cutoff value of 14.1 cm/s with 50% sensitivity and 90.6% NPV, and the flow volume had 0.682 AUROC under the best cutoff value of 201 mL/min with 70.7% sensitivity and 93.5% NPV.

The Plt/Spl showed 0.639 AUROC under the best cutoff value 635.7 with 72.7% sensitivity and 95.3% NPV. There was no significant difference in the AUROC between LGV diameter-based assessment, which was the best in the LGV-related data and the Plt/Spl-based assessment (*p* = 0.377).

## Discussion

The LGV is a major collateral vessel linked to the EV anatomically and hemodynamically. To the best of our knowledge, the current study may be the first to demonstrate the substantial effect of sonographic findings in the LGV as a noninvasive test to diagnose EV. The major advantage of our study is the same-day comparison of sonographic parameters with the endoscopic appearances and blood samples, because the portal hemodynamics may vary according to the patient condition, leading to the potential bias of the data.

First, the positive detection of the LGV strongly suggested the presence of large EV with 100% sensitivity and 100% NPV in the compensated cirrhosis cohort as well as in the whole cohort. Lower LGV detectability in patients without large EV was probably due to the poor collateral development. Next, the hemodynamic findings in the LGV were closely associated with the degree of EV, and were independent of the presence of PHG or gastric fundal varices. However, the diameter-based diagnostic abilities to identify large EV were superior to velocity- or flow volume-based assessments. This would be explained by the LGV having another role as non-variceal collaterals, without forming EV or paraesophageal route; therefore, velocity and flow volume may be susceptible to individual hemodynamic conditions. Detailed anatomical findings regarding the relationship between LGV branches and the lower esophagus may help to provide the differentiation between the LGV linked and that unlinked to the EV. An improvement of detectability in the deeper area and of the signal-to-noise ratio with the US equipment is expected to make this procedure possible without using endoscopic ultrasonography^19^.

A previous study examined the effect of Doppler parameters to diagnose EV, showing only a trend for a higher pulsatility index of the hepatic artery in patients with EV^8^. However, the parameters in the study were hemodynamics in the portal vein, superior mesenteric artery, hepatic artery, and splenic artery, which are non-specific to EV. The advantage of our study is the use of the LGV being specific to EV, which accounts for the significance of the data logically and theoretically. There was another interesting issue, that is, no EV was detected in patients who had LGV with a diameter greater than 10.9 mm, a velocity greater than 19.25 cm/s, or a flow volume greater than 851 mL/min. One of the reasons may be a suppressive effect of portal pressure by the development of advanced collateral vessel^20,21^, and the LGV itself played the suppressing role in such cases.

Our study also used a comparative data, the Plt/Spl reported by Giannini et al^7^. However, the diagnostic abilities are not as high as in the original report, which were 86.7% sensitivity and 93.9% NPV to identify large EV with the cutoff value of 909 presented by their study. Even with the best cutoff value of 442.9 proposed by our study, we found a 0.658 AUROC with 62.1% sensitivity and 87.1% NPV to identify large EV. Although the AUROC showed no significant difference with the LGV diameter-based assessment, sensitivity and NPV seem better in the LGV-based data. The reason may be different patient population with different etiologies, showing an influence on the spleen size which might affect Plt/Spl^22^.

As for the noninvasive US-based markers to identify EV, elastography has gained the most attention^23–26^. A recent meta-analysis study has shown an 87% sensitivity and 53% specificity for EV and 86% sensitivity and 59% specificity for large EV^27^. Although the application of transient elastography (TE) for the spleen appears to offer better ability^28,29^, the data are still insufficient for predicting EV, and the replacement of endoscopy by TE alone may not be encouraged at present because of the low specificity. These TE-based data appear comparable to ours, suggesting the role of sonographic LGV assessment as the important option for the noninvasive assessment of EV.

There are some limitations to our study. First, the study did not examine hepatic venous pressure gradient as a marker for portal pressure; therefore, the severity of portal hypertension under the pathogenesis background for LGV hemodynamics remains unclear. The second limitation is that the assessment of LGV findings depends on the vascular detection. The overall detectability of the LGV was 71.6%, which was affected by the physical size of the patients. However, although EV was positive in 38.5% of the cases with negative LGV detection in our study, none of them had large EV. Since there may be a role in the left gastric artery for the initial formation of EV^30^, the next issue should also be focused on the arterial hemodynamics to identify small EV. Third, the study did not take the inter-operator coefficient variation and/or variability for Doppler parameters into account. The operation by the experienced single physician supports the reliability of the data; however, lack of an evidence of reproducibility may limit the value. In addition, the LGV detectability in the Western patients who tend to have a higher BMI remains undetermined. Therefore, this Doppler-based test would need external validation with multiple operators who may greatly increase the validity of the data.

In conclusion, this same-day comparison study clearly demonstrated the substantial effect of an LGV-based test without needing blood sample to identify large EV with higher sensitivity and NPV in the compensated group as well as in the whole cohort in patients with cirrhosis. It would be quite beneficial to identify patients who are candidates for prophylactic therapy with the noninvasive technique at a much lower cost.

## Study Highlights

### WHAT IS CURRENT KNOWLEDGE

- Noninvasive test for esophageal varices (EV) offers great benefit in patients with cirrhosis.
- The left gastric vein (LGV) is the major pathway for EV.

### WHAT IS NEW HERE

- The sonographic detectability of LGV was 89.6 % in patients with EV.
- Positive detection of LGV showed 100% sensitivity and 100% NPV to identify large EV.
- AUROC values for large EV were similar between LGV-based assessment and Plt/Spl.

### TRANSLATIONAL IMPACT

- The use of ultrasound makes it possible to identify patients with large esophageal varices who are candidates for prophylactic therapy at a much lower cost and noninvasively.
